# Different Biomechanical Cell Behaviors in an Epithelium Drive Collective Epithelial Cell Extrusion

**DOI:** 10.1002/advs.202401573

**Published:** 2024-09-18

**Authors:** Lakshmi Balasubramaniam, Shreyansh Jain, Tien Dang, Emilie Lagoutte, René Marc Mège, Philippe Chavrier, Benoit Ladoux, Carine Rossé

**Affiliations:** ^1^ Université Paris Cité CNRS, Institut Jacques Monod Paris F‐75013 France; ^2^ Wellcome/Cancer Research UK Gurdon Institute Cambridge UK; ^3^ Institut Curie CNRS, UMR144 PSL Research University Paris 75005 France; ^4^ Present address: Transgene S.A. Illkirch–Graffenstaden France; ^5^ Department of Physics Friedrich‐Alexander Universität Erlangen‐Nürnberg 91058 Erlangen Germany; ^6^ Max‐Planck‐Zentrum für Physik und Medizin 91054 Erlangen Germany

**Keywords:** cell sorting, collective cell extrusion, dewetting, mechanobiology, Proc Natl Acad Sci USA

## Abstract

In vertebrates, many organs, such as the kidney and the mammary gland form ductal structures based on the folding of epithelial sheets. The development of these organs relies on coordinated sorting of different cell lineages in both time and space, through mechanisms that remain largely unclear. Tissues are composed of several cell types with distinct biomechanical properties, particularly at cell‐cell and cell‐substrate boundaries. One hypothesis is that adjacent epithelial layers work in a coordinated manner to shape the tissue. Using in vitro experiments on model epithelial cells, differential expression of atypical Protein Kinase C iota (aPKCi), a key junctional polarity protein, is shown to reinforce cell epithelialization and trigger sorting by tuning cell mechanical properties at the tissue level. In a broader perspective, it is shown that in a heterogeneous epithelial monolayer, in which cell sorting occurs, forces arising from epithelial cell growth under confinement by surrounding cells with different biomechanical properties are sufficient to promote collective cell extrusion and generate emerging 3D organization related to spheroids and buds. Overall, this research sheds light on the role of aPKCi and the biomechanical interplay between distinct epithelial cell lineages in shaping tissue organization, providing insights into the understanding of tissue and organ development.

## Graphical abstract

During the formation of ductal structures in organs like the kidney and the mammary gland, mechanisms governing the coordinated sorting of different cell lineages in both time and space remains poorly understood. This research reveals the pivotal role of the key polarity protein atypical Protein Kinase C iota (aPKCi) and the biomechanical interplay between distinct epithelial cells in shaping complex biological structures.

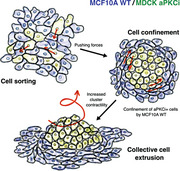

## 1 Introduction

Many organs in vertebrates (breast, lungs and kidney) are composed of epithelial tissues organized in tubular structures.^[^ ^1^ ^]^ The development of these tissues is achieved through the regulated sorting of different cell lineages over time and space.^[^ ^2^ ^]^ Several mechanisms have been proposed to underlie cell sorting events required during organogenesis, including differences in cell‐cell adhesion^[^ ^3^, ^4^, ^5^, ^6^, ^7^, ^8^ ^]^ cell‐substrate adhesion,^[^ ^9^ ^]^ surface tension,^[^ ^10^ ^]^ and nematic activity^[^ ^11^ ^]^ between distinct cell populations, and more recently, cell surface fluctuations.^[^ ^12^ ^]^ However, little is known about how mechanical forces transmit between cells within a given tissue and how cell sorting drives tissue layer formation, and ultimately organ development.

Atypical Serine/Threonine protein kinases, PKC zeta (aPKCz), and iota/lambda (human/mice nomenclature, aPKCi) are the most abundant PKC family members in mouse pre‐implantation embryos^[^ ^13^ ^]^ with aPKCi being indispensable for embryonic survival. Deletion of aPKCi in mice results in embryonic lethality at day 9.5.^[^ ^14^, ^15^ ^]^ aPKCi also plays a central role in the polarization of outer cells of the morula^[^ ^16^, ^17^ ^]^ and promotes the maturation and sorting of the primitive endoderm and epiblast cells into separate layers.^[^ ^2^ ^]^ Interestingly, aPKCi is enriched in “the future” primitive endoderm cells before their sorting from epiblast cells, prior to cell polarization.^[^ ^2^ ^]^ All together, these findings suggest the upregulation of aPKCi expression as a hallmark of epithelialization in the primitive endoderm prior to cell sorting and cell fate determination. A morphogenetic function of aPKC has been described in different organs.^[^ ^18^, ^19^ ^]^ Anisotropic localization of the apical polarity protein, Crumbs, and aPKC between the placode and surrounding cells of the drosophila salivary gland is also required for their invagination.^[^ ^18^ ^]^ In mammals, the mammary gland is an organ made of alveoli and ducts,^[^ ^20^, ^21^ ^]^ the latter composed of an inner layer of luminal cells surrounded by myoepithelial (basal) cells contacting a basement membrane that separates the ductal epithelia from the surrounding stroma.^[^ ^22^ ^]^ During development, branching and invagination of the mammary gland lead to numerous morphological changes.^[^ ^20^, ^23^ ^]^ aPKC and its partner Par3 are essential for end bud remodeling and progenitor differentiation during mammary gland morphogenesis.^[^ ^19^ ^]^

Using MCF‐10A cell multicellular spheroids, a well‐established mammary cell epithelial model, differential expression of aPKCi in individual cells was sufficient to trigger cell sorting.^[^ ^24^ ^]^ Using a mouse mammary organoid transplantation assay, we found that aPKCi overexpression (aPKCi^+^) in a limited number of epithelial cells surrounded by normal cells triggers their basal extrusion in vivo. Our findings further supported that differences in mechanical properties of aPKCi^+^ cells versus normal surrounding cells control the direction of cell extrusion.^[^ ^24^ ^]^ In the present study, we performed in vitro experiments using simple, well‐established epithelial cell models to determine how differential expression of aPKCi can tune the mechanical properties of epithelial cell population, trigger cell sorting and induce tissue rearrangement.

## 2 Results and Discussion

**Figure 1:**
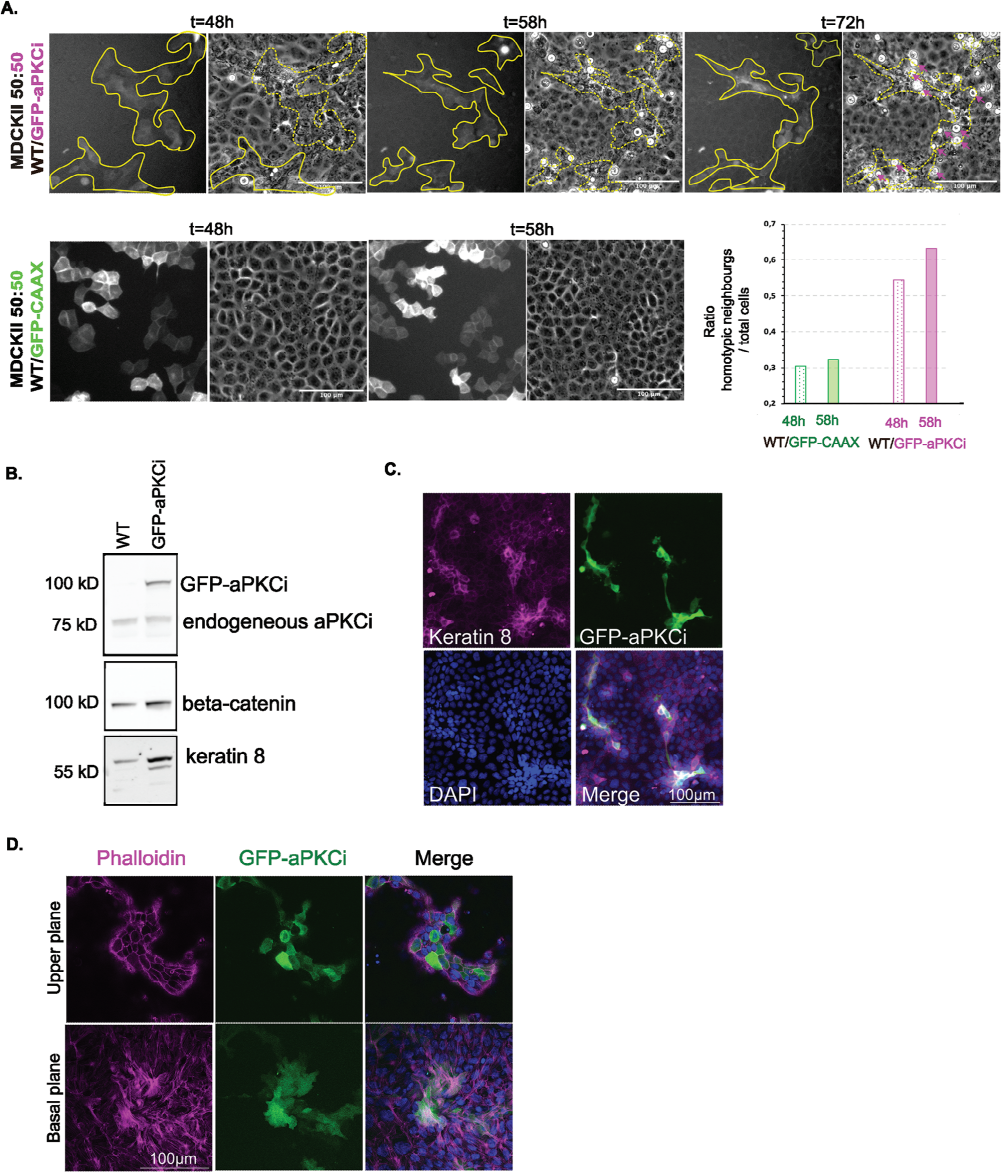
aPKCi overexpression in MDCK cells promotes the expression of epithelial marker keratin 8 and their segregation from the WT counterparts. (A) Phase contrast and fluorescent imaging of MDCKII‐WT mixed 50:50 with MDCKII‐GFP‐aPKCi (top) or with MDCKII‐GFP‐CAAX (bottom) 48 h after seeding. (Scale bar, 100 µm). Extrusions are pointed with pink arrows. On the right, the mixing index was obtained from two independent experiments for PKC and one experiment for GFP‐CAAX. (B) Expression levels of Keratin 8, endogeneous aPKCi, the exogeneous GFP‐aPKCi in MDCKII and MDCKII‐GFP‐aPKCi cells obtained from western blot. Beta‐catenin is the loading control. (C) z‐projection of confocal images of a monolayer of MDCKII‐WT cells and MDCKII‐GFP‐aPKCi cells after 5 days of coculture on glass stained for nuclei (DAPI in blue), keratin 8 (magenta) (Scale bar, 100 µm). (D) Confocal images of a monolayer of MDCKII‐WT cells mixed with MDCKII‐GFP‐aPKCi cells on glass after 3 days of coculture stained for nuclei (DAPI in blue) and actin (phalloidin in magenta). (Scale bar, 100 µm).

We used a classical epithelial cell model, MDCKII cells grown on a 2D glass substrate, to determine if aPKCi overexpression is sufficient to trigger segregation from cells with endogenous aPKCi level. MDCKII‐cells with inducible GFP‐aPKCi expression (MDCKII‐aPKCi) were mixed with wild‐type (MDCKII‐WT) cells at different ratios. When mixed in a 50% ratio, MDCKII‐aPKCi cells segregated away from MDCKII‐WT cells (**Figure** **1**A; Movie S1, Supporting Information), whereas the control MDCKII cells expressing the plasma membrane reporter, CAAX‐GFP, mixed with the MDCKII‐WT cells (Figure 1A; Movie S2, Supporting Information). Furthermore, we observed extrusions of single MDCKII‐aPKCi cells from the MDCKII‐WT monolayer while cells expressing CAAX‐GFP were not extruded (Figure 1A, extrusions indicated with pink arrows / Movies S1 and S2, Supporting Information). MDCKII‐aPKCi cells displayed an increase in keratin‐8 expression in comparison to surrounding WT cells (Figure 1B/C) suggesting a reinforcement of epithelial cell features upon increased aPKCi expression, as previously observed in other models.^[^ ^2^, ^25^ ^]^ MDCKII‐aPKCi cells also presented an increase in basal actin stress fibers when compared to surrounding WT cells (Figure 1D).

Our recent work highlighted that cell sorting can be triggered by differences in nematic (extensile VS contractile) behaviors in the two‐cell populations of the monolayer.^[^ ^11^, ^26^ ^]^ We thus analyzed the nematic behavior of MDCKII‐WT and MDCKII‐aPKCi and found both cell populations were extensile (Figure S1A, Supporting Information(11)). Therefore, differential nematic behavior could not explain in a simple way the cell sorting events between MDCKII‐WT and MDCKII‐aPKCi. Given the morphogenetic potential of aPKCi,^[^ ^16^, ^17^, ^18^ ^]^ we postulated that aPKCi overexpression may have more impact on tissue rearrangement in the context of differential nematic properties. We thus searched for different cell lines presenting a contractile nematic behavior and chose the epithelial mammary cell line MCF‐10A WT (Figure S1A, Supporting Information) that was combined with extensile MDCKII‐aPKCi cells. We further characterized the difference in mechanical features of MDCKII‐aPKCi versus MCF‐10A cells by analyzing their velocity and traction forces in pure cell populations. MDCKII‐aPKCi cells migrated slightly faster than MCF‐10A as analyzed by particle image velocimetry (PIV) (Figure S1B–D, Supporting Information). Traction force microscopy (TFM) revealed that MDCK‐aPKCi cells applied significantly higher forces on the substratum than MCF‐10A cells. When MDCKII‐aPKCi cells were dense and stationary (jammed state) after ≈30 h (Figure S1D, Supporting Information), we observed an increase in the force applied on the substrate (Figure S1E/F, Supporting Information). Altogether these results show a difference in the mechanical properties of MCF‐10A and MDCKII‐aPKCi monolayers (nematic behavior, velocity and forces applied on the substrate).

**Figure 2:**
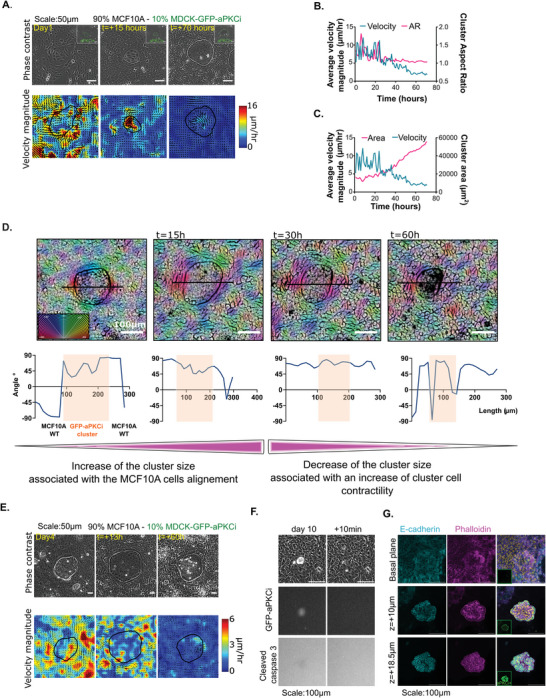
Collective delamination of highly epithelial aPKCi+ cluster is governed by cell sorting and epithelium confinement A) Evolution of cell sorting of MCF‐10A (90%) and MDCKII‐GFP aPKCi (10%) over time represented by phase contrast images (top) and velocity magnitude overlaid with velocity vectors (bottom) at early time points. Marked ROIs represent outline of the aPKCi+ cluster (Scale bar, 50 µm). B) & C) Evolution of aspect ratio B) and cluster area C) (magenta) and cluster velocity magnitude (cyan) over time for 1 example. D) (top) orientation maps overlaid on phase contrast images where the orientation maps refer to the angle of each pixel coarse grained over 10 pixels over different time points (t = 0, 15 h, 30 h and 60 h). (bottom) The angle plotted over the line drawn on the top corresponding image for each image. Orange shaded region indicates the MDCKII‐GFP‐aPKCi cluster and regions outside indicate WT cells. Scale: 100 µm. Color coding of the angles are provided as an inset E) Evolution of cell sorting of MCF‐10A (90%) and MDCKII‐GFP‐aPKCi (10%) over time represented by phase contrast images (top) and velocity magnitude overlaid with velocity vectors (bottom) at late time points. Marked ROIs represent outline of the aPKCi+ cluster (Scale bar, 50 µm). F) Evolution after 10 days of co‐culture of cell sorting of MCF‐10A (90%) and MDCKII‐GFP‐aPKCi (10%) over time represented by phase contrast images (Scale bar, 100 µm) stained for cleaved caspase 3. Delamination of the MDCKII‐GFP‐aPKCi is observed. G) Confocal images of spheroids‐like structure of MDCKII‐GFP‐aPKCi cells on glass stained for nuclei (DAPI in yellow), E‐cadherin (cyan) and actin (phalloidin in magenta). The small inset shows the MDCKII‐ GFP‐aPKCi cells (Scale bar, 100 µm).

We then investigated the impact of these differences in mechanical features on cell sorting and the fate of mixed cell populations. Extensile MDCKII‐aPKCi cells were combined with contractile MCF‐10A cells in a 1‐to‐10 (10%) (**Figure** **2**A; Movie S3, Supporting Information) and 1‐to‐5 (20%) ratio (Figure S1G and Movie S4, Supporting Information). At 1‐to‐5 ratio, (MDCKII‐aPKCi VS MCF‐10A), the two cell populations were sorted into distinct domains, aPKCi^+^ cells took over and compressed MCF‐10A cells, which aligned along the boundary between the two cell populations (Figure S1G and Movie S4, Supporting Information). In contrast, at 10%, the segregation of the two cell populations led to the formation of MDCKII‐aPKCi cell clusters surrounded by MCF‐10A cell domains (Figure 2A and Movie S3, Supporting Information). The same phenotype was observed at 5%.

Mitomycin treatment indicated that segregation was independent of cell proliferation (Figure S2A and Movies S6 and S7, Supporting Information). Altogether, these findings provide insights into the sorting between different epithelial cells, which changes according to the ratio between the two epithelial cell populations and their nematic behaviors.

We then analyzed the dynamics of the mixed cell populations at a 1‐to‐10 ratio. The two cell populations were sorted into distinct domains with distinct velocity profiles (Figure 2A; Figure S1G and Movie S4, Supporting Information). As time progressed, the motility of the surrounding MCF10A WT cells decreased a lot faster than confined MDCKII‐aPKCi cells (Figure 2A). Initially MDCKII‐aPKCi clusters expanded anisotropically, then eventually became more circular as shown by the evolution of the aspect ratio of the clusters, which reached a +1 value after 24 h and then stabilized (Figure 2B; Figure S2C; Supporting Information). Following the rounding up of clusters, MDCKII‐aPKCi continued to proliferate until stabilization of the cluster area and to a lower extent of cell density (Figure S2B, Supporting Information). While the velocity of MDCKII‐aPKCi cells strongly fluctuated during the anisotropic growth phase of clusters, it decreased with time in the isotropic (round) MDCKII‐aPKCi clusters (Figure 2B/C; Figure S2D, Supporting Information). This decrease in velocity was accompanied by an increase in cell density (Figure S2B, Supporting Information). As the MDCKII‐aPKCi cluster size increased, MCF‐10A cells surrounding the clusters progressively aligned tangentially to the edge of the cluster (Figure 2D; Figure S2E and Movies S3,S7, Supporting Information). Interestingly, MCF‐10A cell alignment was not observed when proliferation was inhibited by mitomycin (Figure S2A and Movies S6,S7, Supporting Information), suggesting that proliferation‐induced confinement was important for the alignment of the surrounding contractile MCF‐10A cells. At later time points, the MDCKII‐aPKCi cell cluster area reached a plateau, and concomitantly, surrounding MCF‐10A cells progressively aligned causing the confinement of MDCKII‐aPKCi cells (Figure 2B/E; Movie S8, Supporting Information). MDCKII‐aPKCi cell clusters upon confinement, delaminated from the MCF‐10A epithelium over a few days and were caspase negative (Figure 2F,G; Movie S9, Supporting Information), resulting in the formation of a spheroid‐like 3D structure which budded out of the epithelium (Figure 2G; Movie S10, Supporting Information). This spheroid‐like structure exhibited an internal lumen reminiscent of morphogenic structures such as ducts and acini (Figure 2G; Movie S10, Supporting Information). The initial cluster size appeared to be important as the delaminating clusters contained fewer cells and were smaller in area than the non‐delaminating ones (Figure S2F/G, Supporting Information). This is in line with our observation that at a lower 1‐to‐10 ratio of MDCKII‐aPKCi cells, segregation of the two cell populations led to the formation of MDCKII‐aPKCi cell clusters surrounded by aligned MCF‐10A cells. Altogether, these observations suggest that cells with differential mechanical properties can segregate resulting in the emergence of delaminated cell mass exclusively composed of polarized epithelial cells.

**Figure 3:**
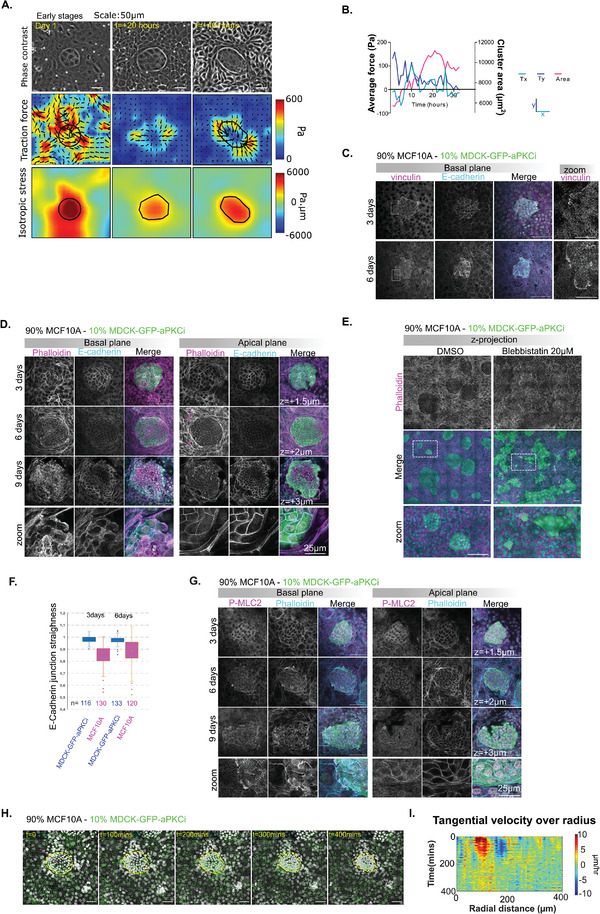
Differential traction forces and stresses govern cell sorting, cluster formation and collective extrusion of MDCKII‐GFP‐aPKCi clusters. A) Phase contrast imaging showing (top layer) cluster evolution with time, traction force magnitude in Pa overlaid with traction force vector (middle layer) and isotropic 2D stress in Pa.µm (bottom layer). B) Evolution of average traction force (Tx, Ty) and area of a single representative cluster (Scale bar, 50 µm). (C/D/G) Confocal images of a monolayer of MCF‐10A cells mixed with 10% MDCKII‐GFP‐aPKCi cells on glass stained for nuclei (DAPI in yellow), E‐cadherin (C/D cyan), and phalloidin ((D/E) magenta, (G) cyan), phospho‐MLC2 (G) and vinculin ((C) magenta) after 3, 6 and 9 days post‐mixing and vinculin ((C) magenta) after 3 and 6 days post‐mixing. Pink arrows in D) show apical actin cables. The height of apical z‐plane from the basal plane is indicated. (Scale bars, 100 µm, zoom scale bars, 25 µm). (E) Mosaic of z‐projection of confocal images of MCF‐10A WT cells mixed with 10% MDCKII‐GFP‐aPKCi cells on glass treated for 2 days with 20 µm blebbistatin or DMSO (control). (Scale bars, 100 µm). F) Quantification of the straightness of the E‐cadherin junctions. n is the number of junctions quantified. (H) Evolution of nuclear movement overlaid with velocity vectors for a cluster of MCF‐10A (90%) and MDCKII‐GFP‐aPKCi (10%) over time. Roi in yellow indicates the GFP‐aPKCi cluster (Scale bar, 50 µm). (I) Kymograph of tangential velocity averaged along the radial direction plotted along the radial direction from center of cluster outward (magenta line in H).

In some tumors, cancer associated fibroblasts (CAFs) align and enwrap the tumor mass actively compressing cancer cells thanks to their actomyosin contractility.^[^ ^27^ ^]^ Similarly, as epithelial MCF‐10A cells align over time around MDCKII‐aPKCi clusters (Figure 2D), we analyzed the forces and stress pattern in these structures to assess whether MCF‐10A cells exert active mechanical stress on MDCKII‐aPKCi cells. We quantified traction forces and monolayer stresses using TFM^[^ ^28^ ^]^ and BISM (Bayesian Inversion Stress Microscopy), respectively.^[^ ^29^, ^30^ ^]^ Expansion of MDCKII‐aPKCi clusters (**Figure** **3**A, 0 h to 20 h) was characterized by inward‐pointing forces exerted by cells on the substratum (Figure 3A, 0–20 h). The sum of vectorial forces underneath MDCKII‐aPKCi clusters approached zero (Figure 3B; Figure S3A, Supporting Information), meaning that the clusters progressively acted as autonomous pluricellular mechanical entities. By computing the stress from traction forces (Figure 3A), we observed a redistribution between 0 and 20 h, and an increase between 20 and 40 h of tensile stress within the MDCKII‐aPKCi clusters that promoted their detachment from the surrounding cells and the formation of a spheroid‐like 3D structure (Figure 3A), in agreement with previous studies on active dewetting.^[^ ^31^, ^32^ ^]^

As cell‐cell adhesion and contractility which enable force transmission^[^ ^33^ ^]^ are key regulators of cell sorting^[^ ^3^, ^9^ ^]^ and buckling,^[^ ^34^ ^]^ we next ask if cell‐cell adhesion and contractility were involved during cluster formation and delamination. Mixed monolayers were immuno‐stained for E‐cadherin, actin, P‐MLC2 (contractility) and vinculin (focal adhesion marker) (Figure 3C–G). In line with the force patterns (Figure 3A/B), we observe increased vinculin staining within the MDCKII‐aPKCi clusters (Figure 3C) as compared to surrounding MCF‐10A cells, in agreement with our previous observations in MCF10A‐aPKCi cells compared to MCF10A‐WT cells.^[^ ^24^ ^]^ Looking at actin organization at the interface between MDCKII‐aPKCi and MCF‐10A cells, we observed the assembly of apical actin cables over time (Figure 3D – pink arrows and Figure S3B, Supporting Information). Such an organization correlated with the emergence of stress patterns showing the compressive state of MCF10A cells surrounding the MDCKII‐aPKCi clusters (Figure 3A, bottom). The formation of these contractile cables, reminiscent of the purse‐string mechanism described during wound healing or cell extrusion,^[^ ^35^, ^36^, ^37^ ^]^ provides a mechanical boundary between the two cell populations. To confirm this hypothesis, we treated the cell mix with blebbistatin (Myosin‐II inhibitor) and observed that the two cell populations mixed again (Figure 3E).

To further investigate the mechanical interaction between the two tissues, we analyzed the distribution of cell‐cell junctions. We observed that the organization of E‐cadherin junctions was different between the two cell populations, with more straight junctions in MDCKII‐aPKCi cells compared to MCF‐10A cells (Figure 3D/F). The alignment of the surrounding MCF‐10A cells over time, causing the confinement of MDCKII‐aPKCi cells (Figure 2C/E/G; Movie S8, Supporting Information), was associated with an increase of E‐cadherin junction straightness of the MCF‐10A‐WT cells around MDCKII‐aPKCi clusters (Figure 3D/F, 3  VS 6 days), suggesting an increase of junctional strength and tension as described.^[^ ^38^ ^]^ However, at the early stages of cell sorting, we detected the presence of E‐cadherin at the interface between MDCKII‐aPKCi and MCF‐10A cells until we observed the separation of MDCKII‐aPKCi clusters from surrounding MCF‐10A cells (9 days) (Figure 3D – 9 days and zoom). We then measured cell velocity within clusters by following the movement of cell nuclei (Figure 3H; Movie S11, Supporting Information). When cluster size stabilized (t = 0 to 300 min) on Day 9, MDCKII‐aPKCi cells generated transitory rotational flows within clusters (as observed in phase contrast, Movies S3, S7 and S9, Supporting Information). This movement resulted in the emergence of shear flows at the MCF‐10A/MDCKII‐aPKCi interface as shown by the tangential velocity profile (Figure 3H,I). This type of movement at the MCF‐10A/MDCKII‐aPKCi interface could potentially destabilize intercellular junctions thereby amplifying the separation between these two epithelia and delamination.

The separation process was followed by the formation of 3D‐like spheroids (Figure 2F / G; Movie S8, Supporting Information). To further understand how the clusters detached from surrounding MCF‐10A cells and formed the 3D structures, we studied the growth of mixed cultures at the later time point (9 days). MDCKII‐aPKCi cells developed actin protrusions enriched in contractility markers visualized by an increase of P‐MLC2 (Figure 3D/G) in combination with an increase of focal adhesions visualized by vinculin staining (Figure 3C). These  changes are supported by our previous observations of an increase in tensile stress patterns within MDCK‐aPKCi clusters. They were  followed by the detachment of the MDCKII‐aPKCi cluster from the surrounding MCF‐10A monolayer, and ultimately by the formation of 3D spheroid‐like structures (Movies S8 and S9, Supporting Information). Importantly, cells in the 3D spheroid‐like structures were alive based on the absence of cleaved caspase 3 staining and polarized as indicated by the presence of a lumen (Figure 2F; Movie S10, Supporting Information).

**Figure 4:**
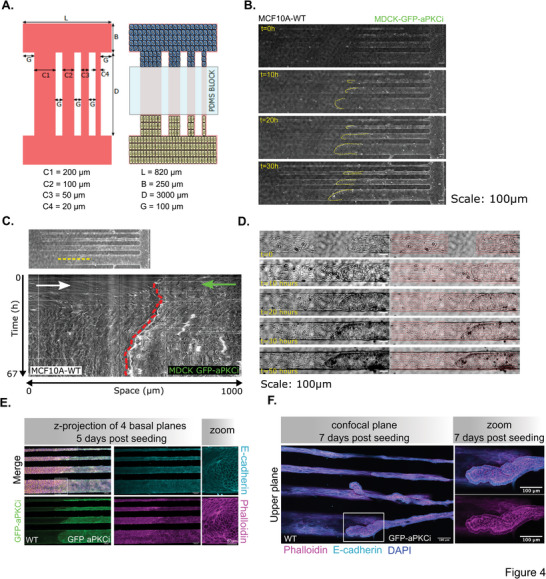
Collision between highly epithelial MDCKII‐GFP‐aPKCi cells with MCF‐10A cells trigger budding of MDCKII‐GFP‐aPKCi cells. A) Schematic of the collision assay. B) Time lapse phase contrast images of the collision assay. (Scale bars, 100 µm). C) Kymograph of the cells before and after collision as a function of time. D) Evolution of the collision between MCF‐10A and MDCKII‐GFP‐aPKCi cells overlaid with orientation vectors over time. Scale bar: 100 µm. E/F) Confocal images of collision between MCF‐10A cells and MDCKII‐GFP‐aPKCi cells stained for nuclei (DAPI in yellow), E‐cadherin (cyan), and phalloidin (magenta) after 5 E) and 7 F) days post‐seeding. (Scale bars, 100 µm).

To better control the interface between the two cell types, we performed a collision assay, in which the two cell types were seeded on two reservoirs connected by fibronectin coated lines of different widths.^[^ ^36^, ^39^ ^]^ After the removal of a PDMS block, cells migrated from both sides until they collided (**Figure** **4**A/B; movie S11, Supporting Information). As the two cell populations met, we observed a transitory regime with a two‐step process: an ingression of WT cells toward aPKCi^+^ cells followed by a reverse movement of MDCKII‐aPKCi cells collectively migrating and pushing onto the MCF‐10A cells for a few hours (Figure 4B,C). Then, as previously described for the MDCKII‐aPKCi clusters, the boundary remained stable over time and aPKCi^+^ cells aligned along this boundary (Figure 4D,E). At later time points, MDCKII‐aPKCi cells also delaminated forming 3D duct‐like structures with a lumen like the ones observed in the 3D mixed cultures (Figure 4F). Altogether, we show that epithelial cells whose growth is confined by another epithelium presenting differential mechanical properties promote collective extrusion leading to the formation of 3D cell masses reminiscent of ducts.

Altogether, we show that epithelial cells overexpressing aPKCi segregate from cells expressing basal aPKCi levels. We show that cell sorting is enhanced by mixing contractile cells with extensile aPKCi‐overexpressing cells. Cell segregation is dependent on contractility but independent of cell proliferation. Epithelial cells overexpressing aPKCi organize into cell clusters that are surrounded by WT cells, and as these clusters grow, contractility within the cluster builds up and leads to cluster detachment. This sequence eventually gives rise to 3D spheroid‐like structures composed of aPKCi overexpressing cells. The whole process is characterized by the establishment of specific force patterns at the interface between the two cell populations, and the emergence of biomechanical structures including focal adhesion, E‐cadherin junctions and contractile actin cables.

We observe that MCF‐10A cells align at the interface with the MDCKII‐aPKCi cell cluster, resisting outward migration under various confining conditions (i.e., varying the ratio between the two cell populations) and in the collision assays. This suggests that the alignment of MCF‐10A cells around MDCKII clusters may function as an “active structure” that hinders the growth and contractility build‐up, ultimately triggering the formation of 3D epithelial cell aggregates.

As cluster size stabilizes, we observe a decrease in the velocity of MDCKII‐aPKCi cells, indicating that they jammed before transitioning into a 3D structure. Meanwhile, MCF‐10A cells align and confine the MDCKII cells within the cluster. Additionally, the differences in P‐MLC2 (contractility) levels between MDCKII‐aPKCi cells and MCF‐10A cells could contribute to the reduction of E‐cadherin at MCF‐10A / MDCKII‐aPKCi interfacial junctions.^[^ ^40^, ^41^ ^]^

The formation of spheroid/duct‐like structures by MDCKII‐aPKCi cells when confined by MCF‐10A cells is in agreement with previous research,^[^ ^34^ ^]^ suggesting that forces generated during epithelial growth under 3D confinement are sufficient to drive folding through buckling. Our findings shed light on aPKCi's function in cell epithelialization and boundary formation ultimately leading to bud emergence. Future studies should tell whether a similar mechanism can occur at the interface between normal and cancer cells or within heterogeneous cancer cell populations.

## 3 Experimental Section

### Cell Culture, Transfection, and Stable Cell Lines

MCF‐10A cells were maintained in DMEM‐F12 (#11039‐021, Gibco) containing 1% penicillin‐glutamine, 10 µg mL^−1^ human insulin (#I9278, Sigma Aldrich), 100 ng mL^−1^ cholera toxin (#C8052, Sigma‐Aldrich), 0.5 mg mL^−1^ hydrocortisone (Sigma Aldrich), 5% horse serum, and 20 ng ml^−1^ EGF (PeproTech) at 5% CO2 in an incubator at 37 °C. MDCK WT (ATCC CCL‐34) cells, MDCK CAAX‐GFP and MDCK‐GFP‐ overexpressing doxycycline‐inducible GFP‐aPKCi cells were cultured in DMEM (containing GlutaMAX, high glucose and pyruvate; Life Technologies) supplemented with 10% fetal bovine serum (FBS; Life Technologies) and 1% penicillin‐streptomycin (Life Technologies) at 37 °C with 5% CO2. pLVX Tet ON GFP‐aPKCiota WT was obtained by the insertion of GFP‐ aPKCiota between the BamHI and AgeI sites of the pLVX Tet ON vector. The construct was checked by sequencing (as described in^[^ ^24^ ^]^). Viral supernatants were generated by transient transfection into the Lenti‐X 293T cell line (Clontech) to infect MDCKWT cells. MDCK‐GFP‐ overexpressing doxycycline‐inducible GFP‐aPKCi were induced with doxycycline at 1 µg mL^−1^ at seeding. In the experiments, when MDCKII‐GFP‐ overexpressing doxycycline‐inducible GFP‐aPKCi and MCF‐10A‐WT are mixed, they are cultivated in MCF‐10A media. The absence of mycoplasma contamination in cell cultures was routinely verified using a PCR test. When needed, doxycycline (Sigma‐Aldrich) was used at a concentration of 1 µg mL^−1^.

### Immunostaining of Cell Monolayers

Cells were counted and mixed with MCF‐10A WT cells at a ratio of 1/10 to 1/20 and seeded at a final concentration ≈500000 cells mL^−1^ on glass coverslips in MCF‐10A medium. To localize E‐cadherin, Keratin 8, P‐MLC2, Actin, and vinculin (antibodies reference:^[^ ^24^ ^]^), cells were fixed in 4% PFA for 20 min and permeabilized h in PBS‐0.5% Triton for 10 min. Blocking was performed in PBS‐10% FBS for 1 h. Primary antibody staining was performed in PBS‐1% FBS‐1%BSA at 4 °C overnight. Secondary antibody incubations were performed for 1 h in PBS. Samples were mounted in Prolong‐DAPI.

### Live‐Cell and Fixed‐Sample Imaging

Long‐term live imaging was performed using a 10 × objective on BioStation IM‐Q (Nikon), a fully automated inverted microscope (Olympus, Japan) and a TI2‐E inverted microscope video NIKON at 37 ° C and 5% CO2. Images are acquired every 10 min depending on a period of 24–72 h. For TFM experiments, phase contrast, and fluorescent beads were imaged simultaneously every 10 min.

Confocal images of MCF‐10A mixed with MDCK‐GFP‐aPKC monolayers on coverslips were acquired with a Leica SP8 NLO microscope equipped with HyD hybrid detectors. Depending on the magnification, various objectives were used (40x oil 1.30 NA). Images were processed using ImageJ software. High resolution imaging was performed using Confocal LSM 980 for MDCK mixed MDCK‐GFP experiments.

Nuclear movement imaging was performed at 37 °C and 5% CO2 with a spinning disk microscope (Gataca‐System) based on a CSU‐W1 Yokogawa head mounted on an inverted Ti‐E Nikon microscope with a motorized XY stage (MadCity Lab). Nuclei was labeled with the Blue DNA dye ENZ‐CHM103‐0200 from Enzo Life Sciences. Images were acquired through a 40 × 1.15 NA with a Photometrics 95B‐sCMOS camera.

### Cell Segmentation

Cell density of 100% and mixed cultures was obtained every hour from live imaging of phase contrast images using Tissue Analyzer.^[^ ^42^ ^]^ Prior to segmentation the images were pre‐processed through contrast adjustment and smooth to ensure noise from cell cytoplasm does not impact cell segmentation. The area and aspect ratio of colonies were obtained through manual segmentation of the contours. Using the segmented mask, the junctions were identified and given a unique id. The cluster area was manually segmented out and calculated over time.

### E‐Cadherin Junction Straightness

E‐cadherin straightness was calculated as the ratio of Euclidean distance over the total distance between two vertices forming the junction. A straightness index of 1 indicates straight junctions while a straightness index < 1 indicates floppy junctions.

### Mixing Index Calculation

The mixing index was calculated as the ratio of homotypic neighbors and the total number of cells.

### Velocity Analysis

Velocities of the monolayer were obtained using PIVlab a Matlab based tool.^[^ ^43^ ^]^ Velocity flow was analyzed using an interrogation window of 82 × 82 and 41 × 41 µm window with a 50% overlap. Erroneous vectors were then filtered out through manual selection and standard deviation filter. Velocity correlation length was calculated using a previously described method.^[^ ^44^ ^]^ The correlation lengths along both directions (x and y along cartesian coordinates) were obtained and averaged to get a final correlation length.

### MSD Calculation

Mean square displacement was obtained as described previously.^[^ ^45^ ^]^ Briefly the instantaneous displacements were first obtained from PIV and MSD was calculated as below:

(1) $$MSD\left(\Delta t\right)=<{\left|r_{i}\left(t+\Delta t\right)-r_{i}\left(t\right)\right|}^{2}>$$

where, *r_i_* (t) indicates the position of a cell I at time t averaged over time and all cells as denoted by <>.

### Nematic Analysis and Orientation Measurements

The formation of nematic defects were detected from phase contrast images as previously described.^[^ ^46^ ^]^ Briefly, orientation was obtained using Orientation J after pre‐processing the image to minimize noise within the cell using a pixel size of 3. Orientation field and defects were detected as described previously. Using winding number parameter, the location of nematic defects were detected within the monolayer. We obtain the local nematic order parameter tensor Q (averaged over a region of 3–4 cells≈90 µm). The largest eigenvector of Q was taken to be the orientation of 2–3 cells and plotted as red lines over the phase image to ensure that the orientation identified is correct. Using this Q value, we automatically detect defect location (+1/2, –1/2, +1 and –1). In order to reduce noise, only stable defects that are found in at least nine consecutive frames (90 min) are used in the following analysis.

The orientation of cells were obtained using OrientationJ using a pixel size of 10 and the average orientation was obtained by drawing a line ROI over the indicated region and the corresponding orientations were plotted.

### Strain Rate Measurement

Defects were first identified as described above and the velocity of the monolayer was obtained as described above. The strain rate was obtained as the gradient of the velocity field as $\dot{\in }=\nabla v$ where v is the velocity. We then plot the strain rate and velocity ≈+1/2 defects by rotating them and aligning them to obtain average plots. Strain rate values were smoothed through linear interpolation.

### Traction Force and Stress Measurements

30 kPa soft silicone substrates were prepared as described previously^[^ ^36^ ^]^ with a CyA and CyB ratio of 1:1.2. Traction forces were obtained by comparing bead displacement against a reference frame which was obtained at the end of imaging by detaching cells with SDS. Upon concatenation of the reference and bead frame, the images were stabilized to avoid drift during imaging, followed by histogram correction to ensure intensities were homogenous across the entire movie. Bead displacement was then obtained using PIV where an interrogation window of 41 × 41 and 20 × 20 µm was used. Forces were then inferred using the FTTC plugin on ImageJ^[^ ^47^ ^]^ using a regularization parameter of 9 × 10^−9^. Stresses were then inferred from traction forces using BISM as described previously.^[^ ^30^ ^]^ The isotropic stress was then computed as half the trace of the stress tensor ((s_xx_+s_yy_)/2). Stress values were smoothed through linear interpolation.

### Collision Assay

Fibronectin (Sigma) patterns^[^ ^26^, ^36^ ^]^ were micro‐contact printed in a 35 mm PDMS (Sylgard 184, Dow Corning) spin coated glass dish. A roughly cut rectangular block of PDMS was then gently placed in the middle of the lines connecting two reservoirs (Figure 4A). The block was cut thin enough so as to act as a barrier between two reservoirs to prevent any mixing while cell seeding. Approximately 0.7 × 10^6^ cells of different types were seeded in the two reservoirs respectively. Cells attached on fibronectin patterns within 45 min. to 1 h. The PDMS block was then gently lifted up and cells were then washed once with DPBS (Dulbecco's phosphate‐buffered saline) (Gibco) and once with DMEM media to prepare the sample for final imaging.

## Conflict of Interest

The authors declare no conflict of interest.

## Supporting information



Supporting Information

Supplemental Movie 1

Supplemental Movie 2

Supplemental Movie 3

Supplemental Movie 4

Supplemental Movie 6

Supplemental Movie 6

Supplemental Movie 7

Supplemental Movie 8

Supplemental Movie 9

Supplemental Movie 10

Supplemental Movie 11

Supplemental Movie 12

## Data Availability

The data that support the findings of this study are available from the corresponding author upon reasonable request.
