# Supplementary material for: Different Biomechanical Cell Behaviors in an Epithelium Drive Collective Epithelial Cell Extrusion
Source: Adv Sci (Weinh). 2024 Sep 18;11(42):2401573. doi: 10.1002/advs.202401573 (PMC11558136; doi:10.1002/advs.202401573)
Supplement: Supplementary file 1 — Supporting Information [file ADVS-11-2401573-s013.pdf]

ADVANCED SCIENCE
Open Access

Supporting Information

for *Adv. Sci.*, DOI 10.1002/advs.202401573

Different Biomechanical Cell Behaviors in an Epithelium Drive Collective Epithelial Cell Extrusion

Lakshmi Balasubramaniam, Shreyansh Jain, Tien Dang, Emilie Lagoutte, René Marc Mège, Philippe Chavrier, Benoit Ladoux* and Carine Rossé**

SUPPLEMENTARY INFORMATION

DIFFERENT BIOMECHANICAL CELL BEHAVIORS IN AN EPITHELIUM DRIVE COLLECTIVE EPITHELIAL CELL EXTRUSION

Lakshmi Balasubramaniam [1,2], Shreyansh Jain [1,3], Tien Dang [1], Emilie Lagoutte [4], René Marc Mège [1], Philippe Chavrier [4#*], Benoit Ladoux [1#*], Carine Rossé [1,4#*]

[1] Université Paris Cité, CNRS, Institut Jacques Monod, F-75013 Paris, France

[2] Wellcome/Cancer Research UK Gurdon Institute, Cambridge, UK

[3] Present address: Transgene S.A., Illkirch–Graffenstaden, France

[4] Institut Curie, Paris Université Sciences et Lettres, CNRS, UMR144, 75005 Paris, France

[#]These authors contributed equally

[*]To whom correspondence should be addressed:

carine.rosse@curie.fr, benoit.ladoux@ijm.fr, philippe.chavrier@curie.fr

Supplementary Figures:

Figure S1

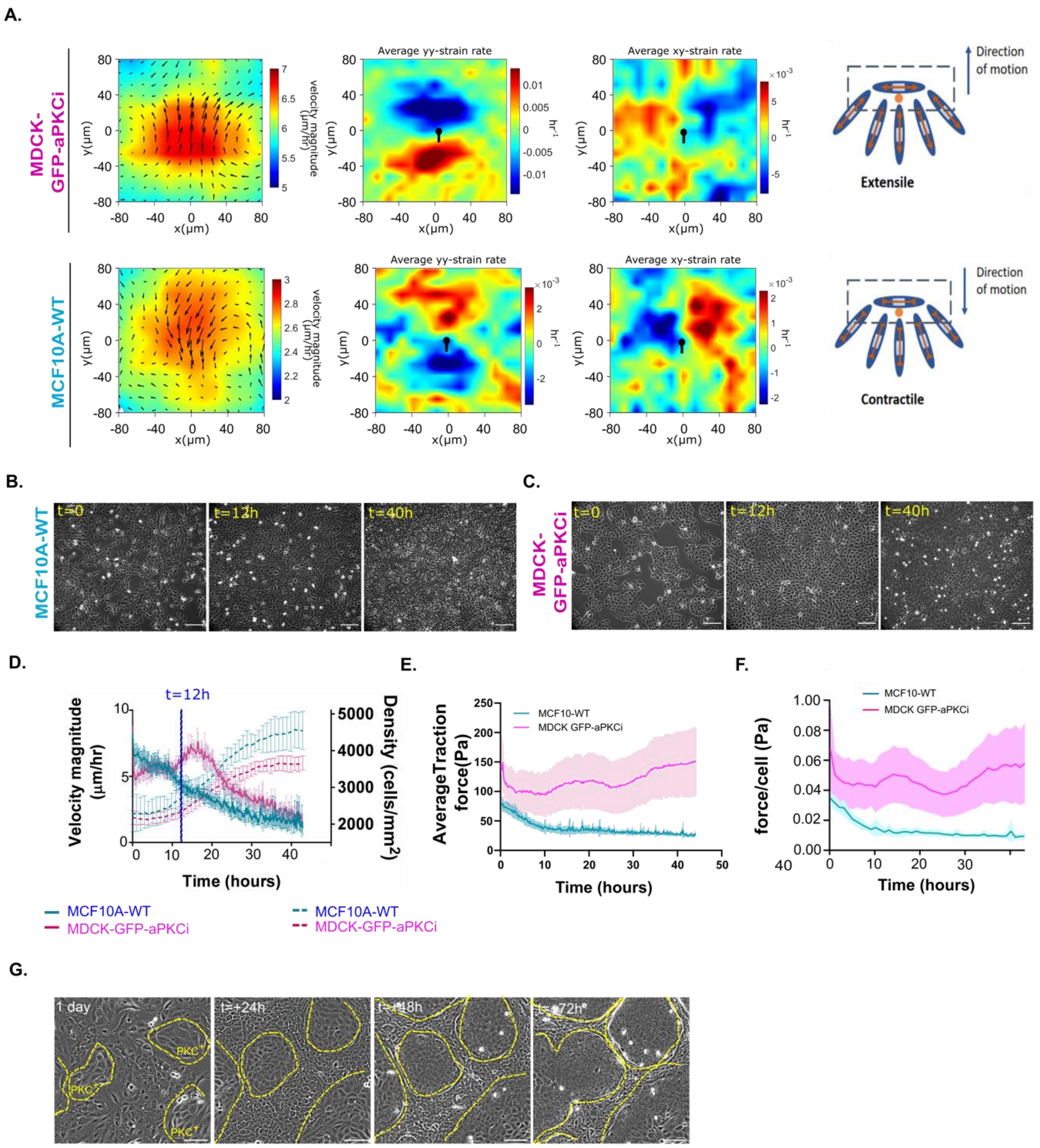

Figure S1: aPKCi overexpression in MDCK cells alters their migratory properties. (A) Averaged velocity magnitude of comet shaped (+1/2) defects (left), average yy strain rate (middle), and average xy strain rate (right) for MDCKII-GFP-aPKCi (top) (n=2592 defects) and MCF-10A (bottom) (n=3182 defects). Schematics on the right show comet shaped defect pattern and the corresponding motion depicting contractile motion for MCF-10A monolayers

(bottom), and extensile motion for MDCKII-GFP-aPKCi monolayers (top). (B/C) Phase contrast imaging of MCF-10A (B) and MDCKII-GFP-aPKCi (C) over time t=0 (left), t=12 hours (middle) and t=40 hours (right). (Scale bar, 100μm). (D) Change of the velocity magnitude (μm/hr) (solid lines) and density (cells/mm^2) (dotted lines) of 100% monolayers of MCF-10A WT (cyan), MDCKII-GFP-aPKCi (magenta) over time. Solid blue line at t=12 hours indicate the time at which confluency is attained obtained from images in Fig. 2A. (F) Evolution of average traction force (Pa) over time for MCF-10A (cyan) and MDCKII-GFP-aPKCi (magenta) monolayers. n is the number of junctions analysed. (G) Evolution of cell sorting of MCF-10A (80%) and MDCKII-GFP-aPKCi (20%) over time represented by phase contrast images. (Scale bars, 100μm).

Figure S2

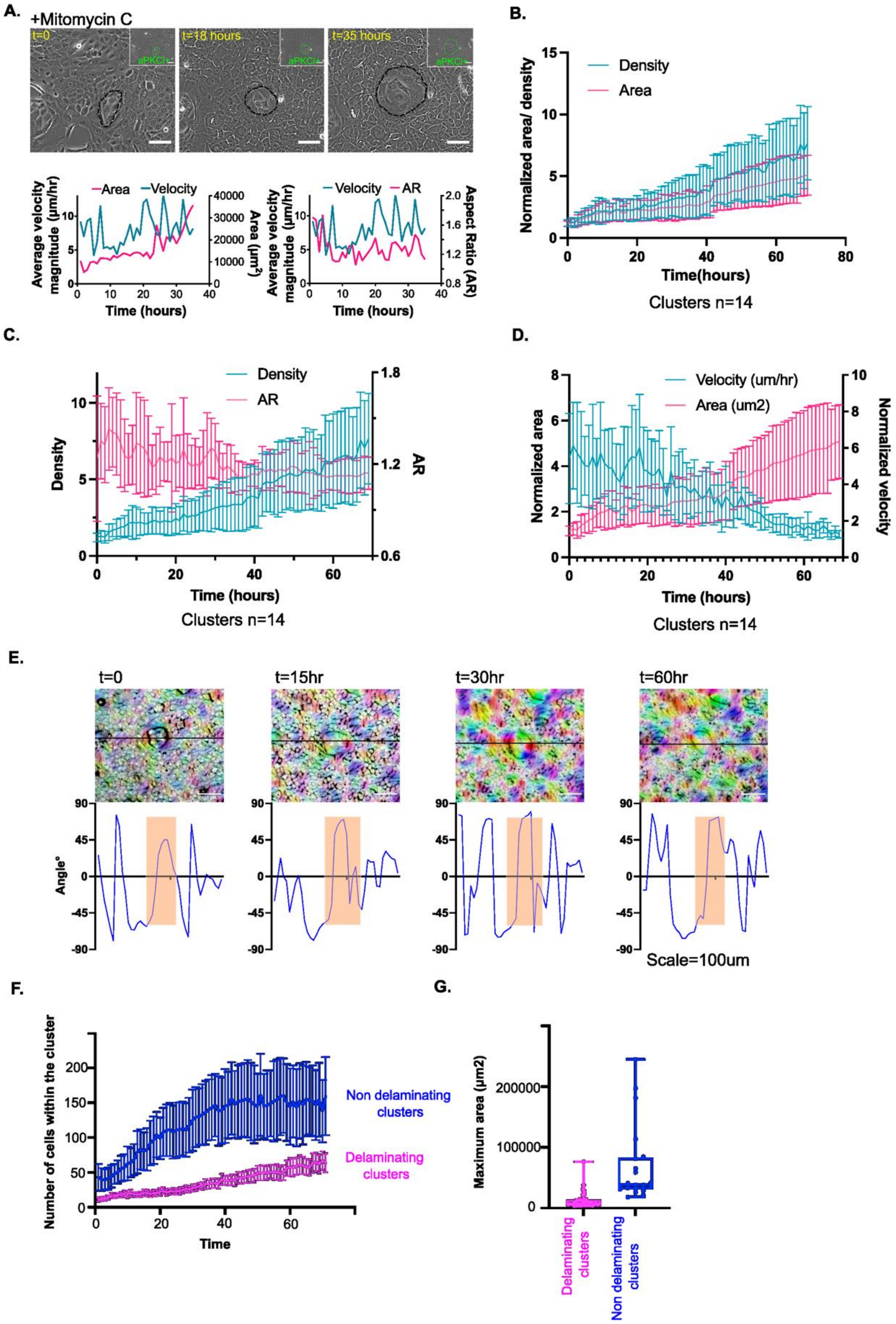

Figure S2: MDCKII-GFP-aPKCi are sorted from MCF-10A independently on cell proliferation but collective cell delamination requires proliferation

(A) Evolution of cell sorting of MCF-10A (90%) and MDCKII-GFP-aPKCi (10%) after treatment of 1h with Mitomycin C (10ng/ml) over time represented by phase contrast images Evolution of cluster area (A - bottom left) / aspect ratio (A - bottom right) (magenta) and cluster velocity (cyan) over time. (B-D) Evolution of normalized cell density (in cyan) with normalized cluster area (B) or aspect ratio (C) in magenta and the cluster area (magenta) with the cluster velocity (cyan) (D) over time for 14 clusters (from 2 independent experiments). (E) (top) orientation maps overlaid on phase contrast images where the orientation maps refer to the angle of each pixel coarse grained over 10 pixels over different time points (t=0, 15h, 30h and 60h). (bottom) The angle plotted over the line drawn on the top corresponding image for each image. Orange shaded region indicates the MDCKII-GFP-aPKCi cluster and regions outside indicate WT cells. Scale: 100μm. (F) Evolution of the number of cells inside the cluster over time according to its fate (delaminating (n=10 clusters from 2 independent experiments) or non delaminating (n=4 from 1 experiment)). (G) Area of 39 delaminating clusters (2 days post confluency) and of 21 non delaminating clusters from 3 independent experiments.

Figure S3

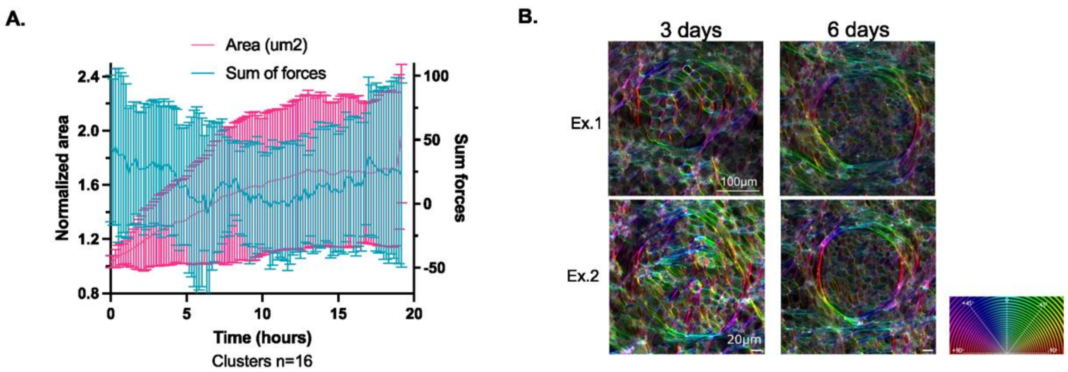

Figure S3: Evolution of the traction forces, the area of the clusters and actin fibers in MCF10A over time.

(A) Evolution of the sum of the traction forces (cyan) in Pa and normalized area of the cluster (magenta) over time for 16 clusters (from 3 independent experiments). (B) Examples of the orientation of actin of monolayer of MCF-10A cells mixed with 10% MDCKII-GFP-aPKCi cells (apical plane) after 3 or 6 days of culture, using image J plugin Orientation J. Error bars represent standard deviation. Error bars represent standard deviation.

Movies:

Movie 1: Time-lapse of MDCKII-WT mixed 50:50 with MDCKII-GFP-a-PKCi. Phase contrast and GFP fluorescent images. Frame every 10min. Scale bar: 100 μm.

Movie 2: Time-lapse of MDCKII-WT mixed 50:50 with MDCKII-GFP-CAAX. Phase contrast and GFP fluorescent images. Frame every 10min. Scale bar: 100 μm.

Movie 3: Time-lapse of a mix of MCF-10A with 10% MDCKII-GFP-aPKCi cells monolayer, one day post-seeding. Phase contrast images. Frame every 10min. Scale bar: 100 μm.

Movie 4: Time-lapse of a mix of MCF-10A with 20% MDCKII-GFP-aPKCi cells monolayer, one day post-seeding. Phase contrast images. Frame every 10min. Scale bar: 100 μm.

Movie 5: Phase contrast images overlaid with orientation vectors obtained from a mix of MCF-10A with 10% MDCKII-GFP-aPKCi cells monolayer. Frame every 10min. Scale bar: 100 μm.

Movies 6 and 7: Time-lapse of a mix of MCF-10A with 10% MDCKII-GFP-aPKCi cells monolayer treated for 1h with Mitomycin C, one day post-seeding. Phase contrast (Movie 5) and GFP fluorescent (Movie 6) images. Frame every 10min. Scale bar: 100 μm.

Movie 8: Time-lapse of a mix of MCF-10A with 10% MDCKII-GFP-aPKCi cells monolayer, four days post-seeding. Phase contrast images. Frame every 10min. Scale bar: 100 μm.

Movie 9: Time-lapse of a mix of MCF-10A with 10% MDCKII-GFP-aPKCi cells monolayer, nine days post-seeding. Phase contrast images. Frame every 10min. Scale bar: 100 μm.

Movie 10: MDCKII-GFP-aPKCi-expressing cells surrounded by MCF-10A cells seeded on a glass coverslip after 10 days post-seeding. The movie corresponds to a z-stack of 3D MDCKII-GFP-aPKCi cluster, showing the localization of nucleus (DAPI, yellow) cleaved caspase 3 (magenta) and ZO-1 (cyan) from the basal plane to the apical plane. The cleaved caspase 3 staining shows that the cells in the cluster are alive. Z-stack confocal images were captured at 1-μm intervals. Scale bar: 20 μm.

Movie 11: Time-lapse of a mix of MCF-10A with 10% MDCKII-GFP-aPKCi cells monolayer, four days post-seeding labelled with DNA dye. Spinning confocal images. Frame every 10min. Scale bar: 100 µm (corresponding to the Fig.3H/I).

Movie 12: Time-lapse of a collision between MCF-10A and MDCKII-GFP-aPKCi cells after few hours after the removal of the PDMS block. Phase contrast images. Frame every 10min. Scale bar: 200 µm.
